# Natural Product Ginsenoside 20(S)-25-Methoxyl-Dammarane-3β, 12β, 20-Triol in Cancer Treatment: A Review of the Pharmacological Mechanisms and Pharmacokinetics

**DOI:** 10.3389/fphar.2020.00521

**Published:** 2020-04-22

**Authors:** Dohyun Kim, Minwoo Park, Iqra Haleem, Younghong Lee, Jain Koo, Young Chae Na, Gidong Song, Jaehwi Lee

**Affiliations:** College of Pharmacy, Chung-Ang University, Seoul, South Korea

**Keywords:** 20(S)-25-OCH_3_-PPD, anticancer activities, molecular pathways, pharmacokinetics, stereoselectivity

## Abstract

*Panax ginseng* has been used as an herbal medicine for thousands of years. Most of its pharmacological effects are attributed to its constituent ginsenosides, including 20(S)-25-methoxyl-dammarane-3β, 12β, 20-triol (20(S)-25-OCH_3_-PPD), which is one of the protopanaxadiol type ginsenosides. It has been found to exhibit anticancer effects by interacting with multiple pharmacological pathways, such as the Wnt/β-catenin, MDM2, ERK/MAPK, and STAT3 signaling pathways. However, its therapeutic potential could be limited by its low bioavailability mainly due to its low aqueous solubility. Thus, several studies have been conducted on its pharmacokinetics and its delivery systems, so as to increase its oral bioavailability. In this review, comprehensive information on its varying pharmacological pathways in cancer, as well as its pharmacokinetic behavior and pharmaceutical strategies, is provided. This information would be useful in the understanding of its diverse mechanisms and pharmacokinetics as an anticancer drug, leading to the design of superior 20(S)-25-OCH_3_-PPD-containing formulations that maximize its therapeutic potential.

## Introduction

The ginseng plant, discovered over 5,500 years ago in China, is a perennial plant of the Araliaceous family, which has been used as a herbal medicine since ancient times (Yun, 2001). Ginseng comprises 14 plants, 12 species, and 2 infraspecific taxa, including *Panax ginseng* C.A. Mey. (Asian ginseng), *Panax quinquefolius* L. (American ginseng), and *Panax japonicus* (T. Nees) C.A. Mey. (Japanese ginseng) (Jia et al., 2009; Shin et al., 2015). It contains medicinally useful chemical constituents with varied structures. Based on their chemical structures, the ginseng produces therapeutic activities through different pharmacological pathways. All ginseng species-derived chemical constituents can be classified into five groups based on their chemical structures such as saponins, polyynes, polysaccharides, flavonoids, and volatile oils (Jia et al., 2009). The main active ingredients of ginseng are the steroidal saponins, which are commonly called ginsenoside (Kim, 2012). They possess a steroid skeleton containing four trans-rings, which have a modified side chain at C-20, and their biological activity is influenced by their sugar binding site. They also differ in the number and site of their hydroxyl groups in their chemical structure (Christensen, 2008; Qi et al., 2010). Another structural difference among them is the stereochemistry at C-20. Most ginsenosides are enantiomeric mixtures of functionally different chemical compounds (Lee et al., 2008; Qi et al., 2010). Chemically, saponins are divided into four groups, protopanaxadiol (PPD), protopanaxatriol (PPT), oleanolic acid type, and ocotillol type (Kim et al., 2017). Both PPD and PPT contain a tetracyclic triterpenoid nucleus in their chemical structure, whereas oleanolic acid possesses pentacyclic triterpene skeleton (Kim, 2012). The main difference between PPD and PPT is the number, position, and type of substituted sugar groups. Sugar moieties in PPD can attach to the C-3 and C-20 of dammarane-type triterpene, whereas sugar moieties in PPT can attach to the C-6, and C-20 (Kim, 2012). The most common ginsenosides in ginseng, including Rb1, Rb2, Rb3, Rc, Rd, Rg3, Rh2, and the aglycone PPD, fall in the 20(S)-PPD classification (Qi et al., 2011).

Ginsenosides are responsible for most of the pharmacological activities of ginseng (Hasegawa, 2004). Some ginsenosides such as Rb1 and Rg1 have demonstrated beneficial effects on central nervous system such as mediating learning and memory processes (Xu et al., 2005). In addition, ginsenosides have shown various pharmacological activities such as hypolipidemic (Kim and Park, 2003), anti-ischemic, anti-arrhythmic, anti-hypertensive (Wang et al., 2007), anti-diabetic (Shang et al., 2007), and hepato-protective activities (Niranjana Murthy et al., 2014). Particularly, they have shown powerful anticancer properties. Ginsenosides affect various pharmacological pathways, including the ERK/MAPK signaling pathway (Qin et al., 2018) and STAT3 signaling pathway(Ai et al., 2017), and in performing their anticancer activity, they down regulate various oncogenic proteins (Bi et al., 2009). Different epidemiological studies have reported that ginseng, as a chemo-preventive compound, lowered cancer recurrence by 50%. Ginseng is also used to improve immune function, which can be helpful for treating cancer as a combinational anticancer therapeutic agent (Chen et al., 2014).

## Pharmacological Significance of 20(S)-25-Methoxyl-Dammarane-3B, 12B, 20-Triol (20(S)-25-OCH_3_-PPD)

Among the known ginsenosides, the anticancer properties of the 20(S)-PPD group are the most extensively studied. 20(S)-PPD has been observed to decrease the proliferation of cancer cells in various *in vitro* and *in vivo* models of breast, colorectal, prostate, hepatic and intestinal cancers (Bae et al., 2004; Gao et al., 2013; Zhang et al., 2018). The presence of sugar moieties in 20(S)-PPD is a very important factor in its anticancer activity, but generally, the anticancer activity of the ginsenosides has been known to decrease as the number of sugar moieties increases (Zhao et al., 2007). This could be due to the polarity of 20(S)-PD, which increases when more sugar moieties attach to it, thereby decreasing its cell membrane permeability (Xu et al., 2003). Additionally, 20(S)-PPD can enhance the anticancer effects of chemotherapeutics by P-glycoprotein inhibition, which prevents the efflux of therapeutically active compounds from tumor cells, making the drugs more bioavailable, and thus, efficiently inducing apoptosis and cell death. 20(S)-PPD selectively inhibits P-glycoprotein expression in tumor cells, thereby reducing the side effects of chemotherapeutics on normal cells (Hao et al., 2008; Ren et al., 2008).

**Figure 1:**
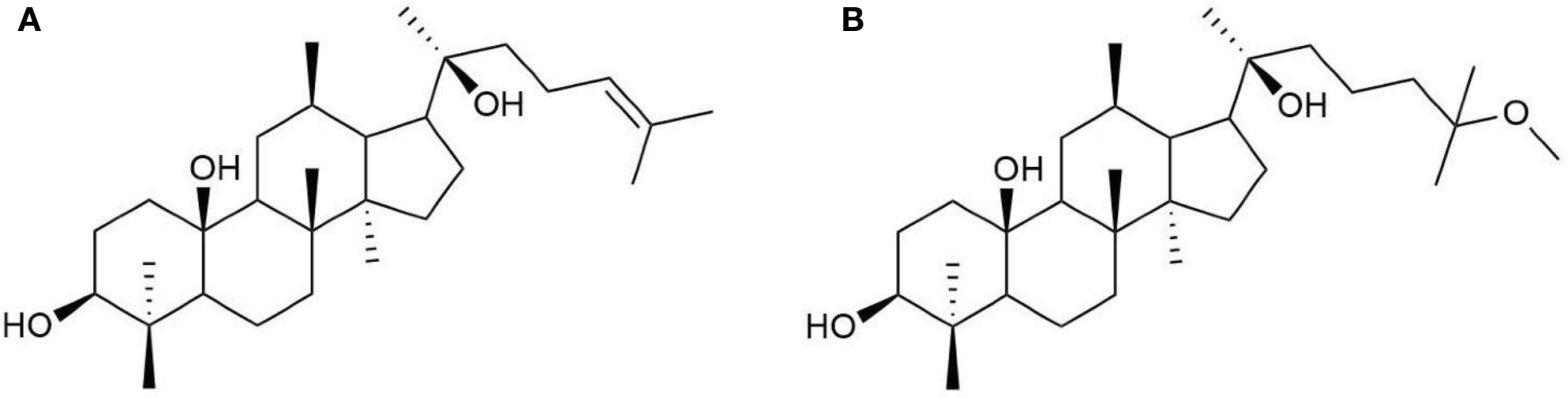
Chemical structure of **(A)** 20(S)-PPD and **(B)** 20(S)-25-OCH_3_-PPD.

20(S)-25-OCH_3_-PPD is a 20(S)-PPD compound, and a tetracyclic triterpenoid with many chiral carbons. It has a methoxy group at C-25 on its side chain, making it different from other 20(S)-PPD compounds that have a double bond between the C-24 and C-25 of their chemical structures as shown in **Figure 1**. Generally, owing to the presence of the methoxy group, 20(S)-25-OCH_3_-PPD has been reported to exhibit better anticancer activities than 20(S)-PPD (Zhao et al., 2007). The half maximal inhibitory concentration (IC 50) value of 20(S)-25-OCH_3_-PPD has been reported to be 2-15 times lower than those of 20(S)-PPD, implying the greater anticancer activity of 20(S)-25-OCH_3_-PPD in most cancer cells than 20(S)-PPD (Zhao et al., 2007). As the previous studies reported that the ginsenosides could be safely used in combination with other chemotherapeutic agents and show enhanced anticancer activity (Wang et al., 2006; Xie et al., 2006), combining 20(S)-25-OCH_3_-PPD with chemotherapeutic agents such as Taxotere and gemcitabine demonstrably exhibited potent anticancer effects (Wang et al., 2008). Tumor growth inhibition was almost complete when combining 20(S)-25-OCH_3_-PPD with either of the two chemotherapeutic agents, Taxotere or gemcitabine. Although the exact mechanism of the anticancer activity of 25-OCH_3_-PPD has not been fully elucidated, many studies have demonstrated that it affects various pharmacological pathways linked to anticancer effects, including the Wnt/β-catenin (Bi et al., 2009), MDM2-p53 (Wang et al., 2012), and ERK/MAPK (Qin et al., 2018) signaling pathways. Through various pharmacological pathways, 25-OCH_3_-PPD can inhibit tumor cell proliferation, induce tumor cell apoptosis, and modulate oncoprotein expression (Bi et al., 2009; Wang et al., 2009).

The chirality of C-20 in 25-OCH_3_-PPD creates two distinct stereoisomers: 20(S)- and 20(R)-25-OCH_3_-PPD, which are different in the orientation of the C-20 hydroxyl group. The chiral inversion from 20(R)- to 20(S)-25-OCH_3_-PPD was not observed after intravenous (IV) administration in rats (Shi et al., 2013). Geometrically, in the S configuration, the C-20 hydroxyl group is close to the C-12 hydroxyl group (Voruganti et al., 2015), and this orientation may provide a better stereoselective interaction for 25-OCH_3_-PPD with lipid membrane of cancer cells, thereby exhibiting stronger antiproliferative and chemopreventive effects (Qi et al., 2010). In addition, 20(S)-25-OCH_3_-PPD has been known to exhibit very low toxicity to non-cancer cells at the same concentration used for treatment of cancer (Lee et al., 2008; Ai et al., 2017). Although many papers have reported the evaluation of the anticancer activities of 20(S)-25-OCH_3_-PPD and the relevant pharmacological pathways, to the best of our knowledge, no review that comprehensively covers the crucial properties of 20(S)-25-OCH_3_-PPD, has been published. Thus, in this review the main pharmacological molecular pathways related to the anticancer activities of 20(S)-25-OCH_3_-PPD are introduced, and its pharmacokinetic properties and recent pharmaceutical strategies are discussed.

## Methods

A comprehensive literature review was conducted after systemic research of “ginsengs, 20(S)-25-OCH_3_-PPD” on PubMed, Web of Science, and Scopus. Seventy-six publications between 2000 and 2019 were included in this narrative review. Out of 76 articles, this review was comprised of 18 original studies. Some other additional studies related to ginsengs but not specifically 20(S)-25-OCH_3_-PPD were also included for the clear picture of 20(S)-25-OCH_3_-PPD pharmacokinetics.

For the better understanding of anticancer activities of 20(S)-25-OCH_3_-PPD, we designed this review into three big sections: pharmacological mechanisms, pharmacokinetics, and pharmaceutical strategies.

## Pharmacological Molecular Pathways Targeted by 20(S)-25-OCH_3_-PPD

### Wnt/β-Catenin Signaling Pathway

**Figure 2:**
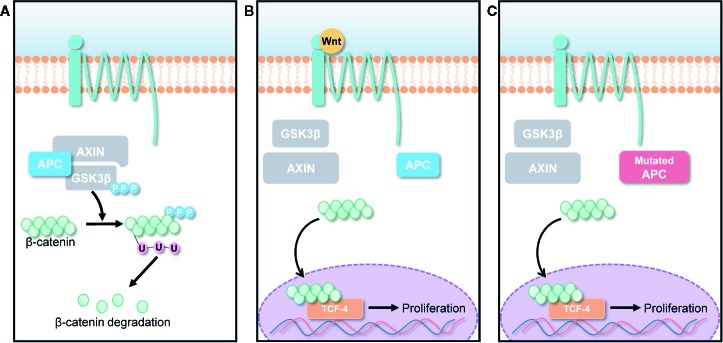
Canonical Wnt/β-catenin pathway. **(A)** In the absence of Wnt signals, β-catenin is degraded by the destruction complex, comprised of Axin, adenomatous polyposis coli (APC), and glycogen synthase kinase 3 (GSK3). Low cytoplasmic β-catenin levels ensure the inactivation of TCF-4, a transcription factor that can promote cancer cell proliferation. **(B)** Overexpression of Wnt ligands and **(C)** APC gene mutation lead to β-catenin accumulation in cancer cells, forming an active complex with transcription factors; thus, promoting cancer cell proliferation.

Wnt/β-catenin pathways are involved in various cellular activities, including cell growth, cell differentiation, and cell survival (Barker and Clevers, 2006; Clevers, 2006). Normally, Wnt signals are absent, and β-catenin is degraded by the destruction complex, composed of Axin, adenomatous polyposis coli (APC), and glycogen synthase kinase 3 (GSK3) as shown in **Figure 2A**. Specific secreted signaling protein such as Wnt protein binds to their frizzled receptor complexes, and activates distinct intracellular canonical or non-canonical Wnt signaling pathways as highlighted in **Figure 2B**. However, β-catenin is overexpressed when a genetic mutation occurs in the APC gene, which is a tumor suppressor gene that controls cell β-catenin levels thereby causing most types of human cancers as described in **Figure 2C** (MacDonald et al., 2009; Yao et al., 2011). Thus, deregulation of Wnt signaling activity and targeting APC gene can be a novel approach for treatment of cancers.

Bi *et al*. evaluated apoptosis and cell proliferation in 20(S)-25-OCH_3_-PPD-treated human colorectal (LS174 and SW480) and lung (A549) cancer cell lines (Bi et al., 2009). Using flow cytometry, they demonstrated that 20(S)-25-OCH_3_-PPD induced cancer cell apoptosis in a dose-dependent manner. In particular, the greatest level of the apoptosis was observed in A549 cell line, whereas LS174 cell line showed the lowest level of the apoptosis. To evaluate the effect of 20(S)-25-OCH_3_-PPD on cancer cell proliferation, they also performed the MTS assay, and their results showed that 20(S)-25-OCH_3_-PPD significantly suppressed SW620 proliferation. Given that β-catenin is the major Wnt/β-catenin pathway signal transducer for modifying different gene expressions *via* transcription factors, they assessed the suppressing effect of 20(S)-25-OCH_3_-PPD on β-catenin expression by evaluating β-catenin transcriptional targets such as cyclin D1, cdk4, and TCF-4, *via* western blotting using cancer cells. The transcriptional targets of β-catenin were significantly reduced in the presence of 20(S)-25-OCH_3_-PPD (Van De Wetering et al., 2002; Bi et al., 2009). This might be because 20(S)-25-OCH_3_-PPD reduced the transcription of the proteins in SW480, A549, and LS174 cells in a dose-dependent manner. Thus, 20(S)-25-OCH_3_-PPD could be used as a potential chemo-therapeutic agent that can induce the apoptosis of cancer cells and inhibit the proliferation of cancer cells (Bi et al., 2009).

### MDM2 Signaling Pathway

**Figure 3:**
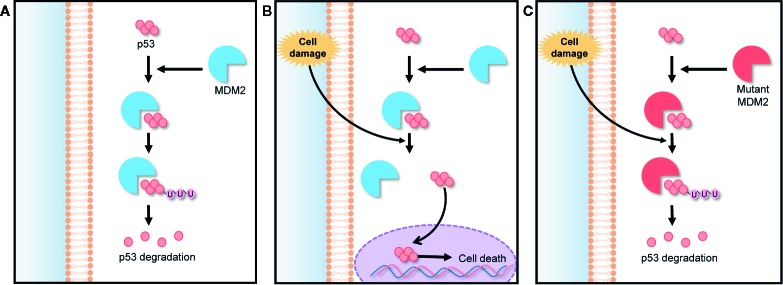
MDM2-p53 signaling pathway; negative feedback loop between p53 and the MDM2 protein in cell cycle regulation. **(A)** In the absence of stress, MDM2 protein binds to p53, keeping it at a low level *via* ubiquitination. **(B)** Under stress, p53 is rapidly stabilized by the breaking of the MDM2/p53 complex, preventing p53 degradation. **(C)** Any MDM2 gene mutation inhibits p53 stabilization under stress, making normal cells more susceptible to oncogenesis.

Murine double minute 2 (MDM2) is a gene that encodes negative regulators responsible for the degradation of p53. The p53 is a tumor suppressor protein encoded by TP53 gene. It regulates cell cycle under stressful situations and plays a critical role in determining whether cells undergo repair or apoptosis, thereby preventing cancer. The cellular level of p53 in the cytoplasm is elevated by a post translational mechanism (Vassilev et al., 2004). The abnormal expression and activity of TP53 gene-encoded proteins can lead to the generation of cancers, including hematologic cancers and some solid tumors (Vassilev, 2007; Tan et al., 2014). The expression of p53 is controlled by an auto-regulatory negative feedback loop involving the MDM2 protein. Under non-stressed conditions, MDM2 binds p53 at its transactivation domain and activates the degradation of p53 by ubiquitination as shown in **Figure 3A**. Owing to this feedback, the cytoplasmic p53 level is kept low. However, under stressed conditions such as DNA damage, hypoxia, and oncogene activation, p53 is rapidly stabilized because the stresses lead to breaking the complex of p53 and MDM2, as shown in **Figure 3B** (Clevers, 2006). Thus, any MDM2 mutation can lead to a loss of this feedback loop control and inhibit the tumor suppressing activity of p53 as demonstrated in **Figure 3C**. The MDM2 mutation also makes normal cells more susceptible to transformation, causing tumor growth (Wang et al., 2012). For this reason, MDM2 is an effective target in many cancer therapies. 20(S)-25-OCH_3_-PPD is involved in the inhibition of MDM2 for protecting p53 degradation and maintaining p53 tumor suppressing function. There are some other pathways those are involved in inhibition of MDM2 by 20(S)-25-OCH_3_-PPD such as ubiquitination of MDM2. MDM2 is also involved in cell cycle progression (Wang et al., 2008). Concisely, 20(S)-25-OCH_3_-PPD is an effective chemotherapeutic agent for treatment of various cancers regardless of p53 status (Wang et al., 2012).

Wang *et al*. performed an *in vitro* wound healing assay to evaluate the effect of 20(S)-25-OCH_3_-PPD on the MDM2 protein and breast cancer metastasis. MDA-MB-231 cells, a breast cancer cell line, were treated with 20(S)-25-OCH_3_-PPD. The MDA-MB-231 cells inhibited cell migration and consequently slowed wound recovery, while the negative control group displayed significantly rapid wound closure, implying that 20(S)-25-OCH_3_-PPD can prevent breast cancer proliferation and metastasis. Western blotting was performed to assess the effect of 20(S)-25-OCH_3_-PPD on MCF7 (wild type p53) and MDA-MB-468 (mutant p53) cells. Mice with tumor tissues were treated with 20(S)-25-OCH_3_-PPD for 5 days each week. After 5 weeks, the tumor tissues were resected from the treated mice, and then the expression levels of MDM2, p53, and p21 ^Waf1/CIP1^ protein in the tumor tissues were analyzed. As a result, levels of p53 and p21 ^Waf1/CIP1^ protein, a primary mediator of downstream cell cycle arrest, increased in the tumor tissue composed of MCF7 cells in a dose and time-dependent manner of 20(S)-25-OCH_3_-PPD. This result implied that 20(S)-25-OCH_3_-PPD induced MDM2 protein inhibition and promoted the activity of p53, thereby suppressing tumor growth. However, in the tumor tissue caused by MDA-MB-468 cells, the level of p53 was not significantly changed because the TP53 gene was mutated in the cells. This result indicated that 20(S)-25-OCH_3_-PPD can inhibit the tumor growth by another mechanism. It has been known that 20(S)-25-OCH_3_-PPD can destabilize MDM2 protein by promoting its ubiquitination and thereby inhibit the tumor growth (Wang et al., 2009). The effects of 20(S)-25-OCH_3_-PPD on the expression of epithelial-mesenchymal transition (EMT) markers, closely associated with tumor growth in human breast cancer cells, were also evaluated. MCF7 and MDA-MB-468 were treated with different doses of 20(S)-25-OCH_3_-PPD for 48 h. As a result, 20(S)-25-OCH_3_-PPD treatment markedly decreased the levels of EMT markers, including twist, vimentin, and snail1 proteins in MDA-MB-231 cells, regardless of the p53 status. For *in vivo* evaluation of the effect of 20(S)-25-OCH_3_-PPD on the breast cancer growth, 20(S)-25-OCH_3_-PPD was administered to nude mice bearing MCF7 xenograft tumors by intraperitoneal injection. After 6 weeks, the size of the tumor tissues was significantly decreased in the mice treated with 20(S)-25-OCH_3_-PPD, whereas the size of tumor tissues in negative control group was not considerably changed. Thus, 20(S)-25-OCH_3_-PPD use can be beneficial in cancer treatment *via* targeting the MDM2 protein (Wang et al., 2012). Another study was also conducted to determine the effects of 20(S)-25-OCH_3_-PPD on both LNCaP (androgen dependent) and PC3 (androgen independent) prostate cancer cells. 20(S)-25-OCH_3_-PPD decreased the expression of MDM2 protein (Wang et al., 2008). This result indicates that 20(S)-25-OCH_3_-PPD decreases cell cycle progression in prostate cancer cells and therefore it could be used as a potent anticancer agent.

### ERK/MAPK Signaling Pathway

**Figure 4:**
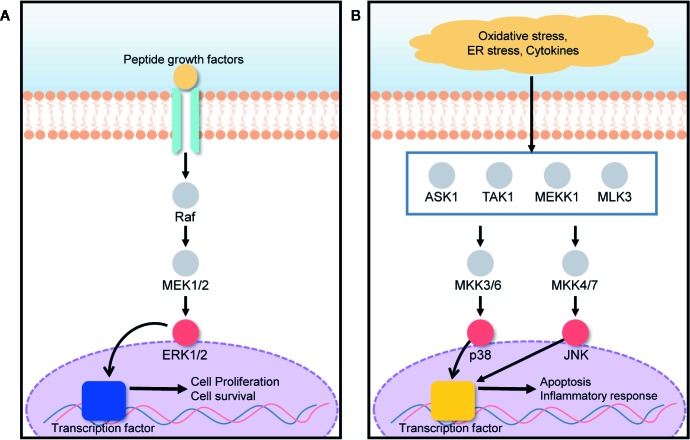
ERK/MAPK signaling pathway is involved in both cell growth and death, based on the cell condition. **(A)** The presence of growth factors activates the ERK1/2 pathway, promoting cell proliferation and survival. **(B)** Under oxidative stress, ER stress, and in the presence of cytokines, MAPK subgroups such as p38 and JNK, are activated, leading to apoptosis and inflammatory responses.

The mitogen-activated protein kinase (MAPK) signal transduction pathway transfers signals from the cell surface to the cell interior for the regulation of cell growth and death. Cell fate is tightly regulated by the MAPK signaling pathway under various stressed conditions such as endoplasmic reticulum (ER) stress, hypoxia, and inflammation (Du et al., 2015; Farrukh et al., 2015). ERK, JNK, and p38 are three MAPK subgroups activated in response to ER stress (Darling and Cook, 2014). The activation and inhibition of the ERK/MAPK signaling pathway depends on the phosphorylation of target proteins such as Ras, Raf, MEK, and ERK (Chung and Kondo, 2011). Under ER stress conditions induced by hypoxia, nutrient deprivation, and a reduction in luminal Ca^2+^ concentration, the protein load in the ER increases, leading to the retention of misfolded proteins within the ER. Consequently, ER stresses activate a series of signaling pathways those are collectively known as the unfolded protein response (UPR) (Healy et al., 2009; Matsuo et al., 2013). Several deleterious diseases are initiated by ER stress. Particularly, liver and kidney cells can be greatly affected by ER stress, leading to hepatocellular carcinoma, acute hepatic failure, glomerular injury, and renal tubule interstitial injury (Kandel-Kfir et al., 2015; Taniguchi and Yoshida, 2015). In the presence of ER stress, ERK1/2 activation, closely associated with the MAPK signal transduction pathway, plays an important role in the protection of cells against UPR-induced cell death, by reducing the load of proteins misfolded in the ER as shown in **Figure 4A** (Jiang et al., 2008; Croft et al., 2014). UPR signaling is responsible for the restoration of homeostasis between folding capacity and protein load, and it can promote cell death in the presence of excessive proteotoxic stress as described in **Figure 4B** (Kim and Choi, 2015). 20(S)-25-OCH_3_-PPD promotes ERK1/2 activation *via* phosphorylation, and correspondingly activates the MAPK signaling pathway for the prevention of ER stress-induced cell death.

Qin *et al*. conducted experiments to demonstrate the effect of 20(S)-25-OCH_3_-PPD on liver and kidney cells (HepG2 and HEK293T cells) in ER stress (Qin et al., 2018). Selenoprotein S (SelS) is a sensitive marker for the evaluation of ER stress (Speckmann et al., 2014). Tunicamycin (TM) was used to induce the ER stress and to increase the SelS expression in HepG2 and HEK293T cells. Reverse transcriptase PCR results showed that 20(S)-25-OCH_3_-PPD signiﬁcantly inhibited the upregulation of TM-induced SelS at the messenger RNA (mRNA) level. Western blotting was also performed to analyze the effects of 20(S)-25-OCH_3_-PPD on the activation of the ERK/MAPK signaling pathway. In this experiment, ERK1/2 phosphorylation and activation were completely inhibited by U0126, to avoid other possible means of phosphorylation. Thereafter, the cells were treated with TM and 20(S)-25-OCH_3_-PPD for 12 h, lysed, and then analyzed using western blotting. It was found that 20(S)-25-OCH_3_-PPD inhibited the up-regulation of TM-induced SelS expression, implying that 20(S)-25-OCH_3_-PPD promoted the phosphorylation of ERK1/2 and thereby protecting cells from ER stress-induced cell death. A cytotoxicity study performed using the MTT assay also demonstrated that the viability of HepG2 and HEK293T cells treated with 20(S)-25-OCH_3_-PPD was not significantly decreased under an ER stress condition compared to those under normal condition without ER stress, indicating that 20(S)-25-OCH_3_-PPD successfully protected the cells from the ER stress condition. These studies highlighted that 20(S)-25-OCH_3_-PPD could activate the ERK/MAPK pathway and reduce the effect of ER stress on liver and kidney cells. Other pathways might be involved in the reduction of ER stress by 20(S)-25-OCH_3_-PPD. However, to the best of our knowledge, the ERK/MAPK pathway plays a leading role (Qin et al., 2018).

### STAT3 Signaling Pathway

**Figure 5:**
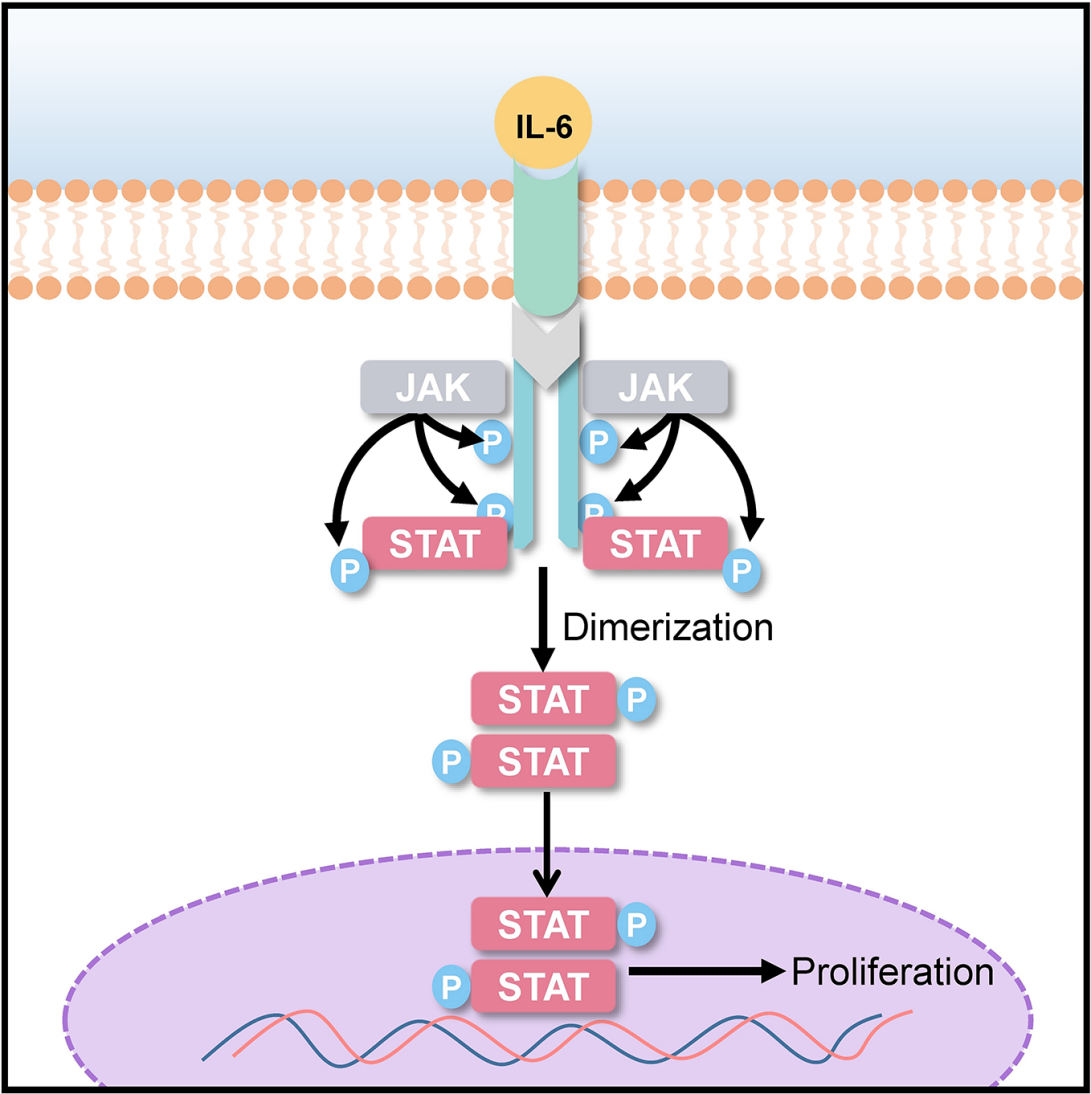
STAT-3 signaling pathway; interleukin (IL)-6 overexpression induces STAT3 phosphorylation, which promotes uncontrolled cancer cell proliferation.

Signal transducer and activator of transcription (STAT) 3 is a transcriptional factor encoded by STAT3 gene. STAT3 imparts signals generated by activated cytokine and growth factor from cell surface receptors to the nucleus for gene transcription. The STAT3 signaling pathway plays a role in cell differentiation, proliferation, apoptosis, angiogenesis, and metastasis, as well as in immune responses (Leeman et al., 2006; Frank, 2007; Quesnelle et al., 2007). It has been observed that STAT3 phosphorylation is elevated in many human cancers and tumor-derived cell lines. Overexpression of the human epidermal growth factor receptor (EGFR) and the family of interleukin (IL)-6 type (IL-6) cytokine receptors lead to the activation of STAT3 as shown in **Figure 5** (Ram and Iyengar, 2001; Wilks, 2008). The activation of STAT3 has been known to cause malignancies, such as hepatocellular carcinoma (He et al., 2013; Ohishi et al., 2014), lung cancer (Songür et al., 2004), and breast cancer (Salgado et al., 2003). Elevated IL-6 level in cancer patients has been associated with poor clinical prognosis (Hong et al., 2007; Pang et al., 2011). For targeted anticancer and anti-inflammatory therapies, IL-6 blocking might prove to be an effective strategy (Hirano et al., 2000). 20(S)-25-OCH_3_-PPD could act as a potential chemotherapeutic agent by inhibiting STAT3 phosphorylation and IL-6-induced STAT3 activation.

For a better understanding of the molecular mechanism of action of 20(S)-25-OCH_3_-PPD in the inhibition of IL-induced phosphorylation, Ai *et al*. established human primary hepatocellular carcinoma (HepG2) xenograft model and divided into multiple treatment and control groups (Ai et al., 2017). The diameters of xenografted tumors were measured before treatment. 20(S)-25-OCH_3_-PPD was intraperitoneally injected once a day/5 days/week. After 4 weeks, mice were sacrificed, and their tumors were weighed and measured (diameters). The result revealed that to an extent, tumor size and mean weight were reduced by 20(S)-25-OCH_3_-PPD treatment. The tumor tissues were then lysed for western blotting, which demonstrated 20(S)-25-OCH_3_-PPD targets in the STAT-3 signaling pathway. At higher concentrations, 20(S)-25-OCH_3_-PPD could directly suppress total STAT3 expression, while at lower concentrations, it significantly inhibited the STAT3 phosphorylation. Thus, 20(S)-25-OCH_3_-PPD could potently block IL-6-induced STAT3 activation in breast cancer cells and HepG2 cells, with very low toxicity to normal liver cells. The MMT assay was performed to check the viability of human breast cancer cells (MDA-MB-231), HepG2, and normal liver cells for 24 h. 20(S)-25-OCH_3_-PPD exhibited stronger cell viability inhibition in cancer cells in comparison to normal liver cells at the same concentration. MMT assay showed that 25-OCH_3_-PPD could be a potential and safe anticancer agent. To evaluate 20(S)-25-OCH_3_-PPD-induced cell cycle arrest, flow cytometry was performed using a six-well plate for 24 and 48 h. According to results, percentage of HepG2 cells in the G1 phase increased while the percentage of S-phase cells decreased, indicating that 20(S)-25-OCH_3_-PPD induced cell cycle arrest in the G1 phase. Together, these results demonstrate that 20(S)-25-OCH_3_-PPD could be a potential anticancer agent.

## Pharmacokinetic Behaviors of 20(S)-25-OCH_3_-PPD

The pharmacokinetic properties of 20(S)-25-OCH_3_-PPD determine its concentration at the site of action and are crucial for its anticancer effect. After its administration, it generally goes through four major pharmacokinetic processes, including absorption, distribution, metabolism, and excretion. Clinical study of 20(S)-25-OCH_3_-PPD has not been any progress so far, but several researches using animal models have been conducted to investigate its pharmacokinetic properties. However, to the best of our knowledge, no review articles thoroughly cover previous studies on its pharmacokinetics. To further enhance 20(S)-25-OCH_3_-PPD bioavailability and anticancer effects, a deep understanding of its pharmacokinetic behaviors is indispensable. Thus, this review also aimed at scrutinizing several studies that investigated its pharmacokinetics, to provide pharmaceutical insight.

**Figure 6:**
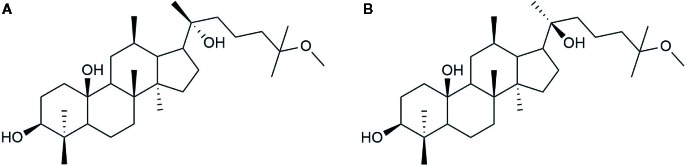
Chemical structure of **(A)** 20(R)-25-OCH_3_-PPD **(B)** 20(S)-25-OCH_3_-PPD.

There are two epimers of 25-OCH_3_-PPD, which differ in configuration at the chiral C-20 as shown in **Figure 6**. Thus, their metabolite 25-OH-PPD, also exists as 20(R) and 20(S) epimers. Understanding the effect of the 20(R/S)-configuration on pharmacokinetics is essential because stereoselective anticancer activity has been observed with 20(R)-PPD and 20(S)-PPD (Qi et al., 2010). 20(S)-PPD has been found to show better anti-proliferative activity than 20(R)-PPD (Qi et al., 2010). Shao et al. investigated the effects of the R/S configuration at C-20, on the pharmacokinetics of 20(R/S)-25-OCH_3_-PPD and 20(R/S)-25-OH-PPD intravenously or orally administered to rats (Shao et al., 2017). The rats were intravenously administered 2.0 mg/kg doses each of 20(R)-25-OCH_3_-PPD and 20(S)-25-OCH_3_-PPD, and their mean plasma concentration-time curves and pharmacokinetic parameters were evaluated. When rats intravenously received 20(S)-25-OCH_3_-PPD, the area under the 20(S)-25-OH-PPD plasma concentration-time curve (AUC) was 2.36-fold higher than that under 20(S)-25-OCH_3_-PPD, suggesting that most 20(S)-25-OCH_3_-PPD epimers were metabolized to 20(S)-25-OH-PPD epimers. However, the AUC_0-t_ value of 20(R)-25-OCH_3_-PPD was 20-fold higher than that of 20(R)-25-OH-PPD, indicating that most 20(R)-25-OCH_3_-PPD epimers were not metabolized, and travelled in rat systemic circulation unchanged. The half-life (t_1/2_) of 20(R)-25-OCH_3_-PPD was determined to be approximately 1 h, which was similar to that of 20(S)-25-OCH_3_-PPD. However, the peak concentration (C_max_), AUC, and the t_1/2_ of 20(S)-25-OH-PPD were 20-fold, 60-fold, and 2-fold higher than those of 20(R)-25-OH-PPD, respectively, after IV administration to rats. Shao et al. suggested two reasons for this stereoselective pharmacokinetics. Firstly, 20(S)-25-OCH_3_-PPD underwent substantial demethylation compared to 20(R)-25-OCH_3_-PPD, implying that it was metabolized to 20(S)-25-OH-PPD to a greater extent compared with 20(R)-25-OH-PPD. Secondly, the pharmacokinetic evaluation of IV administered 20(R/S)-25-OH-PPD subsequently demonstrated that 20(R)-25-OH-PPD was metabolized more efficiently and eliminated faster than 20(S)-25-OH-PPD, leading to its lower concentration in rat plasma compared with 20(S)-25-OH-PPD. Summarily, although intravenously administered 20(S)-25-OCH_3_-PPD was mostly metabolized to 20(S)-25-OH-PPD, the C_max_, AUC, and t_1/2_ of 20(S)-25-OH-PPD were substantially higher than those of 20(R)-25-OH-PPD. As 20(S)-25-OCH_3_-PPD and 20(S)-25-OH-PPD have been reported to present the most anticancer therapeutic effects *in vivo* when comparing the anticancer activities of 20(S)-25-OCH_3_-PPD, 20(S)-25-OH-PPD, 20(R)-25-OCH_3_-PPD, and 20(R)-25-OH-PPD (Zhao et al., 2007; Wang et al., 2008), we could expect that 20(S)-25-OCH_3_-PPD, intravenously administered, would be utilized for further development of anticancer drug, based on its pharmacokinetic data.

With respect to oral administration, 20(R)-25-OCH_3_-PPD and 20(S)-25-OCH_3_-PPD were both orally administered to rats at a dose of 20 mg/kg, and their pharmacokinetic parameters and absolute bioavailability were evaluated. The results showed that 20(R)-25-OCH_3_-PPD reached a C_max_ of 6.18 ± 2.56 ng/ml at 2.2 h. However, the C_max_ of its metabolite 20(R)-25-OH-PPD, could not be measured because it was below the lower limit of quantitation of the LC-MS/MS analysis (5.0 ng/ml). The C_max_ of 20(S)-25-OCH_3_-PPD and its metabolite 20(S)-25-OH-PPD, were determined to be 661.3 ± 240.8 ng/ml at 2.6 h and 2,470 ± 553 ng/ml at 3.6 h, respectively. Thus, the C_max_ and AUC of 20(S)-25-OCH_3_-PPD were 100-fold higher than those of 20(R)-25-OCH_3_-PPD, indicating stereoselective pharmacokinetics in rats after oral absorption. Moreover, the absolute bioavailability of 20(R)-25-OCH_3_-PPD was 0.14 ± 0.19%, while that of 20(S)-25-OCH_3_-PPD was 28.9 ± 13.9%. The reason behind this observation remains unclear; thus, the authors made inferences from other studies that investigated other 20(S)-ginsenosides with geometrical structures like that of 20(S)-25-OCH_3_-PPD (Shao et al., 2017). Reportedly, 20(S)-ginsenosides have a three-dimensional arrangement of hydroxyl groups at the stereocenter (C-20), making them inaccessible to water molecules and more hydrophobic than 20(R)-ginsenosides (Gu et al., 2010; Qi et al., 2010). As a result, they show better membrane permeability than 20(R)-ginsenosides. Although the absorption of orally administered 20(S)-25-OCH_3_-PPD was considerably greater than that of 20(R)-25-OCH_3_-PPD, the low oral bioavailability is still the major drawback of 20(S)-25-OCH_3_-PPD. To address this problem, more studies need to be done on the efficient delivery of 20(S)-25-OCH_3_-PPD.

Several tissue distribution studies have been conducted. Ding et al. evaluated the concentration of 20(R)-25-OCH_3_-PPD in the tissues of various organs after oral administration (Ding et al., 2018). The results showed that 20(R)-25-OCH_3_-PPD was greatly distributed in the liver, lungs, stomach, intestines, female genital organs, and pancreas. Another study also performed tissue biodistribution analysis in CD-1 mice after a 100 mg/kg oral dose of 25-OCH_3_-PPD to evaluate its nanoparticle formulation efficiency (Voruganti et al., 2015). The results showed that the accumulation of 25-OCH_3_-PPD was almost equal in most of the tissues, including the liver, lungs, kidneys, spleen, pancreas, and fat, with a maximum level of 10 ng/g, while its accumulation level was considerably low in heart and brain tissues. Thus, 25-OCH_3_-PPD can be successfully applied to anticancer therapy, including liver, lung, pancreatic, breast, and gastric cancer therapies, with adequate concentrations in these organs. Gender-related differences were observed in the pharmacokinetic behaviors and tissue distribution of 20(R)-25-OCH_3_-PPD and 20(R)-25-OH-PPD in rats, with female rats showing higher plasma concentrations of 20(R)-25-OCH_3_-PPD than male rats after both oral and IV administrations. Additionally, the distribution of 20(R)-25-OCH_3_-PPD in various organs was greater in female rats than that in male rats. Therefore, for the development of 25-OCH_3_-PPD-containing anticancer drugs, further studies should consider not only distribution differences in various tissues but also the gender-related differences in 25-OCH_3_-PPD distribution.

After distribution, the next major step in drug pharmacokinetics is the metabolism of drugs in metabolic organs such as the liver (Shao et al., 2017). Identifying the metabolism routes and metabolites is essential to drug discovery because these properties considerably affect their effectiveness and toxicity. 25-OCH_3_-PPD has been found to undergo extensive metabolism in the human body, and its metabolized forms are known to show lower anticancer effects (Ding et al., 2019). Most of 25-OCH_3_-PPD has been known to be metabolized by the phase 1 metabolism, which largely occurs in the liver *via* CYP3A (Shao et al., 2017). This was demonstrated using ketoconazole, a CYP3A inhibitor (Zhang et al., 2014). When human hepatic microsomes were simultaneously treated with ketoconazole and 20(R)-25-OCH_3_-PPD, 20(R)-25-OCH_3_-PPD concentration in the hepatic microsomes increased, whereas the concentration of its metabolites decreased, implying that CYP3A is a major enzyme for the metabolism of the ginsenoside.

**Figure 7:**
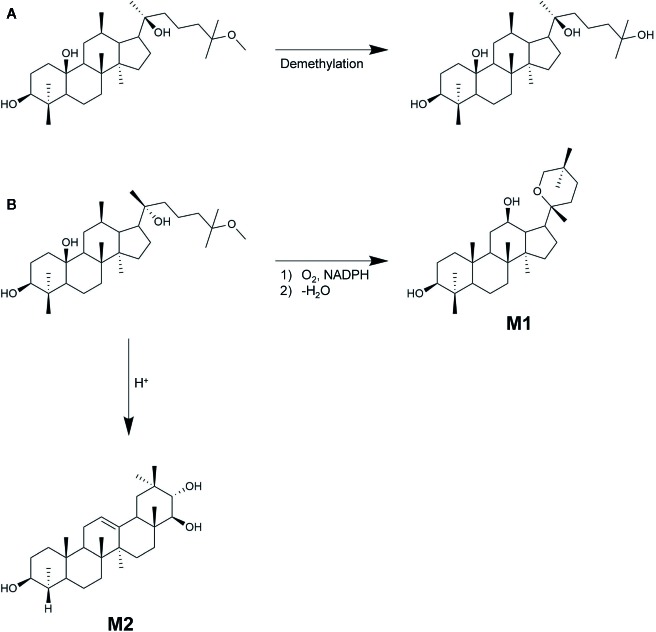
20(S/R)-25-OCH_3_-PPD metabolites. **(A)** 20(S)-25-OCH_3_-PPD metabolized to 20(S)-25-OH-PPD. **(B)** 20(R)-25-OCH_3_-PPD metabolized to 20(R)-PD (M1), and the oleanane triterpenoid saponin M2.

The metabolic fate of 25-OCH_3_-PPD was also investigated by Shao et al. (2017). They demonstrated that the metabolic fate of the ginsenoside was mainly determined by the stereochemistry of its C-20 hydroxyl group. When 20(S)-25-OCH_3_-PPD (0.20 μM), potassium chloride (10 mM), magnesium chloride (10 mM), and 1 mM NADPH in 50 mM potassium phosphate buffer (pH 7.4) incubated in rat liver S9 fraction at 37°C for 30 min, the remaining amounts of 20(S)-25-OCH_3_-PPD and 20(S)-25-OH-PPD were 79 ± 3.18 and 25.22 ± 1.95%, respectively. The total recovery value, which is the sum of the aforementioned values, was 105.0 ± 5.0%, indicating that 20(S)-25-OCH_3_-PPD was mostly metabolized to the demethylated form by CYP3A as shown in **Figure 7A**. On the other hand, the remaining amounts of 20(R)-25-OCH_3_-PPD and 20(R)-25-OH-PPD were evaluated to be 41.77 ± 5.66 and 7.23 ± 0.78%, respectively, resulting in a total recovery value of only 49.00 ± 5.15%, suggesting that 20(R)-25-OCH_3_-PPD might be metabolized by pathways other than demethylation. It has been demonstrated that the most dominant compound of the 16 metabolites of 20(R)-25-OCH_3_-PPD is not 20(R)-25-OH-PPD, but M1, identified as 20(R)-PD as shown in **Figure 7B** (Ding et al., 2019). The reason for this stereospecific metabolism remains unclear, but these differences could be explained using other ginsenosides with the C-20 geometrical arrangement same to that of 25-OCH_3_-PPD. 20(S)-ginsenosides have intramolecular hydrogen bonds between the C-20 and C-12 hydroxyl groups that could increase their stability and form a compact structure (Kang et al., 2005). However, 20(R)-ginsenosides have few intramolecular hydrogen bonds, resulting in the compound showing a more tertiary structure than 20(S)-ginsenosides.

Among the 16 metabolites of 20(R)-25-OCH_3_-PPD, the three major metabolites include M1, M2, and M3, the oxidized form of M2. Based on experimental evidence, M1 and M3 showed insufficient anti-proliferative activity to cancer cell lines compared with 20(R)-25-OCH_3_-PPD (Ding et al., 2019). In contrast, M2, an oleanane triterpenoid saponin, exhibited improved anticancer efficacy in terms of inducing apoptosis and inhibiting cancer cell proliferation. These experiments indicated that the treatment of cancer cells with M2 induced apoptotic morphologies by up-regulating the levels of cleaved caspase-9, caspase-3, and PARP. Additionally, the anti-proliferative activity of M2 in MCF-seven cell lines was 4.90-fold greater than that of 20(R)-25-OCH_3_-PPD.

25-OCH_3_-PPD elimination behavior was also evaluated in rat models, such as clearance, half-life, and excretion. After 20(R/S)-25-OCH_3_-PPD was intravenously administered to rats at a dose of 2.0 mg/kg (Shao et al., 2017), the clearance of 20(R)-25-OCH_3_-PPD and 20(S)-25-OCH_3_-PPD were 2.45 ± 0.59 and 1.70 ± 0.50 L/h/kg, respectively. This difference in clearance might be due to differences in their hepatic metabolism rates. The R-isomer showed a faster hepatic metabolism rate than the S-isomer (Shao et al., 2017). Although the authors demonstrated the clearance profiles of 20(R/S)-25-OCH_3_-PPD after IV administration, their excretion behaviors could not be perfectly assumed using the clearance data (Toutain, 2004). Ding *et al*. assessed the excretion of 20(R)-25-OCH_3_-PPD after oral administration to rats at dose of 20 mg/kg (Ding et al., 2018), which might be helpful to expect that of S-isomer. The ginsenoside was found to be mainly excreted *via* feces, urine, and bile, and excretion peak was shown at 12–24 h after oral administration to rats. There were also some differences in the urinary and fecal excretion of 20(R)-25-OCH_3_-PPD and its metabolites between female and male rats. With respect to urinary excretion, the recovery cumulative excretion (RCE) of 20(R)-25-OCH_3_-PPD in female rats was 2.64-fold higher than in male rats. This difference in urinary 20(R)-25-OCH_3_-PPD excretion may result from gender differences in renal clearance, including filtration, secretion, and reabsorption. Regarding fecal excretion, male rats showed a considerably higher RCE (8.507 ± 2.339%) than that of female rats (0.288 ± 0.185%). However, there were little differences in the RCE of bile and feces (IV administration), and blood 20(R)-25-OCH_3_-PPD concentrations were significantly higher in female rats than in male rats. These results suggested that the absorption differences between gender might cause the differences in fecal excretion. Summarizing these data, female rats showed a higher urinary excretion rate and a similar fecal excretion rate (IV administration) compared to male rats. Consequently, the absorption rate and the half-life of 20(S)-25-OCH_3_-PPD are higher than those of 20(R)-25-OCH_3_-PPD (Shi et al., 2013; Shao et al., 2017). Therefore, 20(S)-25-OCH_3_-PPD might be more advantageously used as a novel anticancer drug compared to 20(R)-25-OCH_3_-PPD.

## Recent Attempts for Delivery of 25-OCH_3_-PPD

Along with pharmacokinetic studies for 25-OCH_3_-PPD, several studies were performed to develop delivery systems improving the oral bioavailability of 25-OCH_3_-PPD, which is the major drawback for the development of anticancer agent. Although the paper focuses on 20(S)-25-OCH_3_-PPD, we comprehensively reviewed the research outcome related to both 20(R)- and 20(S)-25-OCH_3_-PPD to give the reader pharmaceutical insights as much as possible

A self-microemulsifying drug delivery system (SMEDDS) was shown to enhance oral bioavailability of 20(S)-25-OCH_3_-PPD (Cai et al., 2014). A SMEDDS is an isotropic mixture of oil, surfactant, and possibly co-surfactant, which forms fine oil in water (o/w) emulsions upon mild agitation when exposed to an aqueous media, such as gastrointestinal (GI) fluids. The optimized SMEDDS formulation for 25-OCH_3_-PPD contained Cremorphor^®^ EL (50%), glycerin (20%), and Labrafil^®^ M1944 (30%) as a surfactant, co-surfactant, and oil, respectively. The 20(S)-25-OCH_3_-PPD-loaded SMEDDS spread readily when exposed to the distilled water and formed fine particles with a mean droplet size of about 40 nm within a minute. In pharmacokinetic study evaluated with oral administration of the SMEDDS and suspension of 20(S)-25-OCH_3_-PPD in rats, the results showed that AUC_0-∞_ measured with the SMEDDS was 9.8 times greater than that assessed with the suspension. The reasons for this improved oral bioavailability were assumed to be enhanced solubility and lymphatic absorption of 20(S)-25-OCH_3_-PPD. Thus, we could find the promise of SMEDDS for enhancing the oral bioavailability of 20(S)-25-OCH_3_-PPD.

A polyethylene glycol (PEG)-poly(lactic-co-glycolic acid) (PLGA) nanoparticle was demonstrated to improve the oral absorption and the anticancer activity of 25-OCH_3_-PPD (Voruganti et al., 2015). The PLGA, approved by Food and Drug Administration for used in therapeutic devices owing to its biodegradability and biocompatibility, is an efficient carrier for the delivery of poorly soluble drugs, and the PEG was used for coating the PLGA nanoparticles. The PEG-PLGA nanoparticles developed in this study showed the average diameters less than 43 nm and were stable at various pH conditions such as pH 7.4, pH 6.8, and pH 1.2. The improved permeability of the PEG-PLGA nanoparticles was demonstrated by employing caco-2 cell line, an *in vitro* model for intestinal epithelial permeability studies. In fact, compared to 25-OCH_3_-PPD alone, the *in vivo* tumor uptake of 25-OCH_3_-PPD was increased when 25-OCH_3_-PPD incorporated in the PEG-PLGA nanoparticle was administered to the nude mice bearing PC3 xenograft tumors. It implies that the PEG-PLGA nanoparticle loading 25-OCH_3_-PPD could be precisely delivered to the tumors. In addition, the pharmacokinetic studies of 25-OCH_3_-PPD performed in male CD-1 mice showed that the C_max_ value of the oral administration of 25-OCH_3_-PPD-loaded PEG-PLGA nanoparticles was nine-fold greater than that of the same oral dose of merely 25-OCH_3_-PPD.

In fact, *in vivo* efficacy study of 25-OCH_3_-PPD-loaded PLGA nanoparticles was conducted employing the PC3 xenograft model of human prostate cancer. After a 4-week oral treatment of 25-OCH_3_-PPD-loaded PEG-PLGA nanoparticles and merely 25-OCH_3_-PPD, the inhibition of the PC3 tumor growth of the treatment group was 87%, whereas that of the control group was merely 41%. Furthermore, there were no any other signs of toxicity which were evaluated by examining mouse body weight and histological findings. Overall, 25-OCH_3_-PPD-loaded PEG-PLGA nanoparticles they prepared was demonstrated to be effective formulations by the pharmacokinetic, efficacy, and safety studies, implying that the nanotechnology-based approaches could be applicable for delivering 20(S)-25-OCH_3_-PPD.

A nanoemulsion system employing phospholipid complexes was designed to increase the oral bioavailability of 20(R)-25-OCH_3_-PPD (Zhang et al., 2016). The phospholipid complexes, the main components of the nanoemulsion system fabricated, have amphiphilic properties so that they can act as surfactant with considerably lower toxicity than the surfactants used for the SMEDDS, in the previous study of Cai et al. (2014), where large amounts of surfactants such as Cremorphor^®^ EL could cause gastrointestinal irritation and be toxic (Gelderblom et al., 2001). Phospholipid complexes with 20(R)-25-OCH_3_-PPD were produced at a different molar ratio and formulated to the nanoemulsion system in 1% PEG400 water solution. As a result, the nanoemulsion system based on phospholipid complexes of 20(R)-25-OCH_3_-PPD (25-OCH_3_-PPD-phospholipid complex) represented the enhanced aqueous solubility of 4.9 times. Furthermore, the AUC_0-24h_ and C_max_ values of 25-OCH_3_-PPD-phospholipid complex were 3.5- and 3.9-fold higher than those of the free compound. The reason for this was that the nanoemulsion system could have large surface area and high cell permeability due to the nano size of droplets of 25-OCH_3_-PPD-phospholipid complex (Shafiq et al., 2007). Therefore, the nanoemulsion system utilizing phospholipid could be usefully exploited as effective and safe delivery system for 20(R)-25-OCH_3_-PPD with enhanced bioavailability.

## Conclusion and Future Perspectives

Multiple studies have been conducted to investigate the potential anticancer effects of 20(S)-25-OCH_3_-PPD. However, to the best of our knowledge, no review articles have comprehensively covered the pharmacological mechanisms and pharmacokinetics of 20(S)-25-OCH_3_-PPD. Therefore, this review attempted to comprehensively summarize the pharmacological pathways involved in tumor progression, including the Wnt/β-catenin, MDM2, ERK/MAPK, and STAT3 signaling pathways, and provide information on prospective 20(S)-25-OCH_3_-PPD molecular targets for cancer treatment. Most of the pharmacological pathways targeted by 20(S)-25-OCH_3_-PPD could show anticancer effects by the three mechanisms, such as inhibiting tumor cell proliferation, inducing tumor cell apoptosis, and modulating oncoprotein expression. Moreover, several studies, which investigated the pharmacokinetic behaviors of 20(S)-25-OCH_3_-PPD in animal models, were thoroughly discussed. Pharmacokinetic studies of 25-OCH_3_-PPD administered intravenously and orally were systemically summarized, indicating that the compound is potential candidate as an anticancer drug lead. More sophisticated delivery strategies for 20(S)-25-OCH_3_-PPD are required to improve the solubility and target precisely the desired site of action. This review would be useful for the pharmaceutical scientists interested in 20(S)-25-OCH_3_-PPD as a promising anticancer agent.

## Author Contributions

Conceptualization: DK, MP, IH, and JL. Methodology: IH, DK, MP, JK, YN, YL, GS, and JL. Writing—original draft preparation: DK, IH, MP, and JL. Funding acquisition: JL. Supervision: JL.

## Funding

This study was supported by a National Research Foundation of Korea (NRF) grant, funded by the Korean government (MSIP) (Grant number, 2015R1A5A1008958). This research was also supported by the Chung-Ang University Graduate Research Scholarship in 2018 (DK).

## Conflict of Interest

The authors declare that the research was conducted in the absence of any commercial or financial relationships that could be construed as a potential conflict of interest.
